# Experimental Counterexample to Bell’s Locality Criterion

**DOI:** 10.3390/e24121742

**Published:** 2022-11-29

**Authors:** Ghenadie N. Mardari

**Affiliations:** Open Worlds Research, Sparks, MD 21152, USA; gmardari@gmail.com

**Keywords:** Bell’s theorem, EPR paradox, quantum entanglement, non-locality, classical superposition, quantum superposition, Malus’ law, joint measurements, correlation

## Abstract

The EPR paradox was caused by the provision that quantum variables must have pre-existing values. This type of “hidden property realism” was later falsified by Bell’s Theorem. Accordingly, the physical basis for action-at-a-distance between entangled quanta was removed. Yet, modern interpretations present Bell’s inequality as a Locality Criterion, as if Bell violations can only happen at the quantum level, and only with remote interactions. This is a questionable practice, considering that classical joint measurements also violate such inequalities for mutually exclusive wave properties. In particular, consecutive measurements of polarization produce the same coefficients of correlation as parallel measurements with entangled quanta, yet they are explicitly local. Furthermore, it is possible to combine parallel and consecutive measurements of Type I polarization-entangled photons in a single experiment, conclusively showing that quantum Bell violations can be local. Surprisingly, classical phenomena also require nonlocal interpretations if pre-existing properties are taken for granted. Hence, the solution is to reject the models with pre-existing properties for both classical and quantum wave-like phenomena.

## 1. Introduction

Quantum observables can be interpreted in two ways. They can be described as *pre-existing* properties of quantum projections, or simply as qualities that are *created* by the act of measurement. Einstein, Podolsky and Rosen (EPR) argued in 1935 [1] that quantum entanglement supports the first alternative. Yet, their proposal was falsified by Bell in 1964 [2] by showing that pre-existing values cannot reproduce the predictions of quantum theory. Jointly distributed variables have limited coefficients of correlation, in a manner that quantum variables do not [3]. Since then, the experimental implications of Bell’s Theorem were decisively confirmed [4,5,6,7,8,9,10,11,12]. Unfortunately, Bell’s solution created a new problem. The physical quality of being “pre-determined” is not equivalent to the statistical quality of being “jointly distributed”. Bell’s Theorem applies in equal measure to predetermined and to non-deterministic properties, as long as they are formally separable from each other. Hence, Bell defined his famous inequality as a Locality Criterion, suggesting that *formally* inseparable quantities correspond to *physically* inseparable measurements. In other words, if Bell’s Theorem is correct, then quantum theory is irreducibly nonlocal [2]. In retrospect, this is a tenuous argument because mathematical accuracy does not necessarily entail interpretive accuracy. Indeed, several authors have recently shown that algebraic separability is a measure of statistical compatibility [13,14,15]. Contextuality does not entail non-locality (See also [16,17], and references therein). In support of this finding, it will be shown below that Bell’s Locality Criterion has a straightforward experimental counterexample.

Just like quantum observables, complex wave profiles can also be interpreted in two ways. They can be described as collections of simple spectral components that act in unison, or as patterns of oscillation that are irreducibly complex. Accordingly, it can be argued that sharp wave measurements reveal preexisting components that are “always there”, or that they simply create new patterns of oscillation by transforming the input profile. On the one hand, pre-existing properties are supported by numerous macroscopic observations and have a strong intuitive appeal. On the other hand, net state approaches are better at predicting the details of wave diffraction. (As a reminder, Huygens’ Principle was famously rescued by the Fresnel correction [18,19]). Yet, they are often perceived as useful tools with limited ontological significance. The problem is that most wave-like phenomena appear equally compatible with both of these interpretations, and—for a long time—it did not seem possible to decide which of them is ultimately correct. Still, given the structural similarities between classical and quantum wavefunctions, it is worth asking: can we use Bell’s Theorem to solve this problem? After all, Bell-type inequalities cannot be violated by preexisting properties, but they may or may not be obeyed by created properties. As shown elsewhere [20,21], this is indeed a promising avenue of research. The two problems can even be described as two sides of the same coin, and arguably fix each other’s shortcomings. Furthermore, the physical significance of various mathematical relationships is easier to determine in classical contexts. For the purpose of this discussion, the relevant implication is that quantum *and* classical wave-like phenomena can finally be explained without paradoxes, as will be shown below.

In what follows, Section 2 will discuss the similarities between classical and quantum measurements of optical polarization and show that consecutive measurements violate Bell-type inequalities with local means. Section 3 will extend this conclusion to parallel measurements and present an experimental counterexample to Bell’s Locality Criterion. Finally, Section 4 will outline the interpretive ramifications of this result, and also show that Bell’s Theorem is more important than previously understood. Instead of acting as a marker for non-classical behavior, it can serve as a tool for unifying the analysis of classical and quantum phenomena. The challenge is to distinguish the models that “interpret the math” from the models that “interpret reality”.

## 2. Bell Violations with Consecutive Classical Measurements

It is often suggested that Bell violations are a special quantum effect, implying that classical projections cannot display the same kind of behavior. Yet, this cannot be true because quantum and classical observables are known to obey the Correspondence principle [22]. Namely, quantum distributions are quantitatively identical to classical distributions for large numbers of detection events. For example, two orthogonal polarizers are able to block a quantum beam completely. In other words, all the photons that pass through a vertical polarizer cannot also pass through a horizontal filter (in ideal conditions). Yet, this rule can be broken by introducing a diagonal polarizer in-between the two original filters. According to Malus’ Law, 25% of the quanta that can pass through the vertical filter will also pass through the horizontal filter in this case. It is very well known that classical optical projections *also* obey Malus’ Law and display *the same* kind of behavior when they interact with polarizing filters or beam-splitters [23]. Yet, if classical projections also obey Malus’ Law, then they also produce incompatible observables with contextual correlations.

Let us consider a classical laser beam, passing through two linear polarizing filters. The first filter has a vertical axis, while the second has a relative angle of 22.5°. According to Malus’ law, 85% of the output from the first polarizer will pass through the second. Next, let us imagine that the second polarizer is rotated even more, to the diagonal plane (45°). The new output is going to be 50%, but the question is: how did this happen? Was the new diagonal projection created by transforming the input laser beam, or did the “measurement” simply reveal a preexisting component? If the diagonal polarization was “always there” in the vertical polarization, then the 22.5° polarization is also “still there”, even though it is no longer observed. However, such a hypothesis is not supported by the details of this experiment, because the three observables have incompatible relationships. In particular, 85% of the output from the 0° filter must be transmitted by a 22.5° filter, and the same relationship holds between the imaginary 22.5° plane and the output 45° plane. This means that the lowest possible proportion transmitted by the diagonal polarizer should be 72% (i.e., 0.85 × 0.85 × 100%), if these three variables are assumed to have joint distributions. Yet, Malus’ Law predicts 50% and this is what is also happening in actual experiments. In short, Malus’ Law predicts polarization observables that cannot be explained with jointly distributed variables. Therefore, classical measurements of polarization must also produce violations of Bell inequalities.

**Figure 1:**
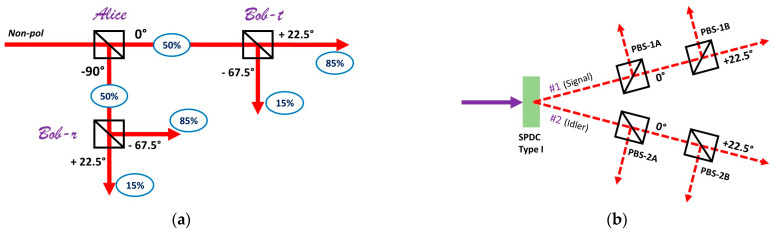
**Bell experiments with consecutive and parallel joint measurements.** Classical measurements of polarization produce the same distributions as quantum measurements, but the interpretation is less ambiguous. (**a**) A non-polarized laser beam is split 50-50 by a polarizing beam-splitter (PBS) designated as “Alice”. A second PBS is added in each output projection, designated as “Bob-t” for the transmitted channel, and “Bob-r” for the reflected channel. The correlations between consecutive measurements of polarization are governed by Malus’ Law, and Bell violations follow by default. (**b**) Identical polarization-entangled photons are produced via Type I spontaneous parametric down-conversion (SPDC). Each beam has an experimental set-up similar to the classical example on left. The quantitative features of this arrangement show that parallel measurements of photon polarization have the same underlying mechanism as the described consecutive classical measurements. Bell violations do not require communication between Alice and Bob.

This conclusion can be verified by conducting a Bell experiment with classical laser beams. The important difference is that parallel measurements must be replaced with consecutive observations in this case. As will be shown below, this has an important interpretive advantage. Hence, a depolarized laser beam can be split 50-50 by a polarizing beam-splitter (PBS), at any angle of measurement. The outcome is a pair of projections with orthogonal linear polarization (e.g., vertical and horizontal, diagonal and anti-diagonal, etc.). If we add another PBS in each output projection (Figure 1a), we can determine the coefficient of correlation between any two consecutive states of polarization. With a suitable choice of four observables, a complete Bell experiment can be performed. The most convenient way to conduct such a test would be to use the measurement scheme proposed by Clauser, Horne, Shimony and Holt (CHSH) [24]. The notable difference in this context is that correlated beams are used instead of anti-correlated quanta. Accordingly, the CHSH inequality for this experiment is:

S = |E(**A**,**B**) + E(**B**,**C**) + E(**C**,**D**) − E(**A**,**D**)| ≤ 2. (1)

As it is well-known, the highest possible violations with optical states of polarization are expected when the four variables are separated by an angle of 22.5°. For example, if variable **A** corresponds to a PBS setting with A_T_ = 0° (and A_R_ = 90°), then the remaining variables can be defined such that B_T_ = 22.5, C_T_ = 45° and D_T_ = 67.5°. As shown in Figure 1a, pairwise measurements can be performed by designating the first PBS as “Alice”, and the second PBS in each output projection as “Bob”. To give just one example, if PBS_Alice_ is aligned to transmit 0° polarizations, then PBS_Bob_ can be aligned to transmit 22.5° polarizations. The Alice PBS always has an output of 50-50, because the input projection is depolarized. The Bob PBS will always transmit and reflect radiation in strict accordance with Malus’ Law. In this case, Bob is predicted to observe an 85-15 split in Alice’s transmitted channel, and a 15-85 split in Alice’s reflected channel. The coefficient of correlation is determined according to the standard rule:

E(**A**,**B**) = (T**_A_**T**_B_** + R**_A_**R**_B_** − T**_A_**R**_B_** − R**_A_**T**_B_**)/100%. (2)

Three joint measurements—E(**A**,**B**), E(**B**,**C**) and E(**C**,**D**)—have the same relative angle of 22.5°. Therefore, they must yield a coefficient of correlation of approximately 0.7. However, the final joint measurement corresponds to an angular separation of 67.5°, and the approximate coefficient of correlation of E(**A**,**D**) = −0.7. As a result, we can see that the four coefficients of correlation produce a violation of the inequality (1) mentioned above:

S = |0.7 + 0.7 + 0.7 − (−0.7)| = 2.8.(3)

Moreover, this is not just any random violation. This is exactly the same result that is predicted by quantum theory for joint measurements with polarization-entangled quanta and confirmed by the celebrated experiments of Aspect and collaborators [4,5,6].

As a corollary of the above, Bell violations can happen for many kinds of wave-like observables, if they do not commute. This cannot be interpreted as a uniquely “quantum” phenomenon. More importantly, consecutive measurements with classical projections reveal several technical details that are not available in quantum experiments with entangled quanta. First of all, we can see that Malus’ Law does not depend on the order of measurements. For any two observables **X** and **Y**, Alice can measure the first and Bob the second, or vice versa, without any effect on the resulting coefficient. Secondly, if Alice makes one measurement, then it is irrelevant which other potential observable is chosen for measurement by Bob. In other words, once Alice makes the first measurement, her contribution is over. The second measurement can be in any of the two chosen orientations, and Bell violations still happen. Therefore, it is not true that Alice and Bob need to know what the other party is going to measure, and their local distributions do not need to change in any way in response to such knowledge. Finally, note again that Bell violations are possible for high intensity classical projections. They can even be replicated with waves on a rope, or other types of classical oscillations that may be polarized. In short, Bell violations are possible for any system that obeys Malus’ law. It is not necessary to invoke metaphysical processes in order to explain this behavior.

Ontologically speaking, Bell violations are telling us that non-commuting measurement outcomes do not correspond to preexisting qualities (be they random or predetermined). A wave can be transformed in many different ways, but classical objects cannot move in several directions at the same time. Therefore, alternative outputs cannot be simultaneously real in the input profile. For example, a rope cannot be shaken simultaneously in the vertical and the horizontal plane. It can only move in the direction of the vector sum of all the forces that act on it. Accordingly, it is misleading to describe polarizing filters as “measurement” devices. They can provide useful information about input beams, but they only do so by revealing what types of transformation are possible. Furthermore, as seen above, Alice makes her measurements before Bob, but this cannot explain the observation of Bell violations. To be a little more precise, Alice’s device may determine the input profile for Bob’s device, but it cannot force it to obey Malus’ Law. Quite the opposite: Bob can choose between different transformations, independently from Alice, but all of those alternatives are locally incompatible with each other. In plain language, the wave propagates until it reaches Alice’s filter. The effect of the filter on the wave is governed by Malus’ Law. Then, the output wave propagates until it reaches Bob’s filter. Malus’ Law applies again. In short, Bell violations emerge as a natural classical effect in this context. Notwithstanding, we can ask: is there an experimental way to prove that Malus’ Law is local? The answer is that we can prepare identical systems and replace consecutive measurements with parallel measurements. If Bell violations still occur, then Malus’ Law does not depend in any way on the interaction between remote measurements devices.

## 3. Interpretive Equivalence of Consecutive and Parallel Measurements

A possible objection to the preceding demonstration is that parallel measurements are substantially different from consecutive measurements, especially in the quantum regime. In a typical Bell experiment, entangled quanta are measured only once. In contrast, consecutive measurements apply several transformations to one and the same entity. In order to address this concern, let us consider a pair of beams with polarization entangled quanta. For simplicity, let us assume that entangled quanta produce identical measurement outcomes, when measured in the same way. (In practice, this can be achieved with Type I parametric down-conversion [25,26]). As shown in Figure 1b, every projection has two polarizers. First, let us consider what happens if both beams have the first PBS oriented at 0° and the second at 22.5°. In each path, the quanta are expected to obey Malus’ Law for consecutive measurements. Yet, the two quanta must always be transmitted at the same time (or reflected at the same time) by the first PBS (1A and 2A, respectively), because they are maximally correlated. This means that some of the measurements across the two beams are interchangeable. In particular, measurement A of quantum 1 will obey Malus’ Law in coincidence with measurement B in its own path, as well as with measurement B in the path of quantum 2. The same is true for quantum 2. Accordingly, we see that parallel measurements across the paths display the same relationships as consecutive measurements along the paths. This is sufficiently explained by the symmetry between the two projections. There is no need to invoke any kind of information exchange between measurement devices for this effect.

Next, we can reverse the order of measurements in the path of quantum 2, so that the angle of measurement at 2B is now applied at 2A, and vice versa. As shown above, the order of observation does not matter, and Malus’ Law is still obeyed. Yet, this means that measurement 1A will display the same coefficient of correlation with the second measurement in its own path (1B) as with the first measurement (2A) in the path of quantum 2. Again, no communication between the two paths is required for this phenomenon. The crucial detail is that measurement 1A can remain fixed, while measurement devices at 1B and 2A can be switched between alternative settings. Malus’ Law is obeyed in any configuration, but PBS-1A is sampling the same random variables at all times. Therefore, it is not true that Alice needs to know what Bob is doing, in order for their joint measurements to violate Bell-type inequalities, even at large distances (and even at the quantum level).

As a corollary of the above, Malus’ Law is better interpreted an intrinsic rule of any polarized system. If an object is “such that” it obeys Malus’ Law for any order of consecutive measurements, then it is also “such that” it obeys Malus’ Law for parallel measurements of polarization with its identical clone. A quantum cannot help but satisfy the prescriptions of Malus’ Law in such measurements. Therefore, it can only behave in this way, and there is no qualitative difference between the two alternative methods for observing the same coefficient. An obvious feature of consecutive measurements is that Alice has a direct impact on the input of Bob. Nonetheless, it is a mistake to interpret this effect as a necessary condition for the manifestation of Malus’ Law. In other words, Alice’s device does not “cause” a quantum to behave according to Malus’ law in Bob’s device. It simply defines the way in which the intrinsic propensity to obey this law would manifest itself in particular observations. Another way to think about this is that any measurement is a transformation. Alice transforms her quantum in a chosen way. If Bob chose to repeat the same exact measurement, Bell violations would not be observed. Yet, Bob can also choose to transform his input beam in two alternative ways. These new observables cannot be reconciled as coexisting properties of Alice’s output projection. This is why Alice and Bob obtain correlations that cannot be explained with jointly distributed variables. In short, it is not Alice’s effect on Bob that determines the violation. It is Bob’s local choice to rotate the polarization profile in opposite directions that produces this effect. Likewise, if Alice and Bob make parallel measurements, the underlying mechanism is not substantially different. Identical inputs with identical transformations produce identical outputs. If Alice makes a measurement, Bob’s quantum is “such that” it would produce the same outcome for an identical transformation. Therefore, it is also “such that” rotating the input state produces output states that obey Malus’ Law, relative to Alice’s anchor state. Intuitively, it may feel as if Alice has to change her behavior in response to Bob’s choice when Bell violations occur, yet this is only true for pre-existing properties.

## 4. Discussion

As suggested at the beginning of this analysis, the physical reality behind binary measurements of polarization is not self-explanatory. When a beam passes through a PBS, it is split into two components. The vector sum of these components is equal to the input vector, but how should we think about the input projection? According to the established tradition in classical wave mechanics, any act of decomposition is supposed to reveal the physical spectrum of pre-existing components of the input beam (rather than its virtual structure). In other words, the observable net profile of any optical projection can be interpreted as an illusion. The “true underlying reality” must consist of various spectral components, acting together without perturbing each other. For example, if a laser beam is measured by a PBS with the fast axis in the vertical plane, then the input beam must have contained two components: one vertical and one horizontal. However, if the PBS is rotated to the diagonal plane, then the input beam contained a different set of pre-existing components: one diagonal, and the other antidiagonal. The problem with this explanation is that classical entities cannot move in two directions at the same time. Yet, the PBS can be used to measure hundreds of different angles of observation. Therefore, it is not possible to explain how all of these alternative “pre-existing” configurations are possible at the same time. In order to maintain a classical description of the underlying reality, new physical factors must be invented, such as to explain the “retro-causal” connection between observable features and the implied input pre-existing features. For this to work, incoming waves must “know” what the measurement setting is going to be and must change their properties all the way back inside the laser. Notice that such nonlocality is needed to explain *individual* alternative measurements, before we even start to consider joint observables. Hence, even if the mechanism behind Malus’ Law is explained with metaphysical processes, having remote measurements does not add anything new the big picture.

Another alternative is to assume that polarization measurements do not reveal pre-existing components, but rather “create” them. In other words, the net state of the incoming projection is assumed to be physically real, while the interaction with the PBS is assumed to produce two new components whose total angular momentum is equal to the input value of the “parent” state. If polarization measurements are interpreted as transformations, then there is no mystery to explain. Output profiles of wave transformations are contextual, and there is no requirement for alternative outcomes to be statistically compatible with each other (unlike pre-existing particle properties). This interpretation is particularly resonant with the principle of “completeness” of quantum theory. If the net state is all that there is, then there are no pre-existing components that correspond to observable outcomes. Yet, this means that popular interpretations of quantum behavior, stating that particles are “in many states at the same time”, or that correlated measurements are “inseparable from each other”, express preconceived notions about classical superposition, and actually contradict the basic features of quantum superposition. Surprisingly, quantum superposition is ontologically classical, and classical superposition is ontologically non-classical.

With this clear distinction between pre-existing and created properties, we can see that Bell’s inequality is a formidable tool, able to settle debates about the nature of wave superposition at any level of observation. In contrast, it fares quite badly as a “test of locality”. For instance, Bell himself argued that his inequality applies to any type of hidden variables, be they deterministic or irreducibly random [2]. Yet, if the values of a random variable have no underlying mechanism, then it is not possible to explain even ordinary correlations between two events. Any pattern of coincidence requires metaphysical (and perhaps “nonlocal”) explanations in such a case, even if Bell’s inequality is obeyed. Therefore, it is not possible to prove that algebraic separability entails locality. Why should it then follow that algebraic *in*separability entails *non*-locality? As a reminder, the EPR argument suggested that non-commuting properties exist at the same time. Then, given that such properties are jointly real, they should be “reasonably” assumed to be pre-determined, or else strange consequences ensue. Though, when Bell acknowledged that his inequality applied to predetermined and to random phenomena in equal measure, he showed that his discovery could not shed any light on the ontological validity of determinism. Therefore, he had no grounds to make any speculations about locality or nonlocality. Indeed, this whole conundrum was rendered moot by his demonstration that non-commuting variables *cannot* be real at the same time.

Another interesting nuance is that algebraic separability (the condition for obeying a Bell inequality) is widely perceived as a relationship between quantum observables. This is why Bell experiments are regularly reported as tests of local realism [4,5,6,7,8,9,10,11,12]. Yet, quantum observables are strikingly well separated. After all, non-commuting properties can only exist one at a time by default. Instead, the property of algebraic inseparability applies to the *input* quantum wave-function. In other words, the *unmeasured* profile cannot be factorized into meaningful components. This is the basis for claiming that measurement results do not express pre-existing properties (and that quantum theory is “complete”). When a set of properties are real at the same time, they can be formally expressed with jointly distributed variables. In this case, they cannot violate Bell-type inequalities [3]. In contrast, when a set of properties are mutually exclusive, they are not constrained to a shared probability space. They can produce unconstrained combinations of properties, and even violate Bell-type inequalities in special cases [13,14,15,16,17]. In short, it makes no physical sense to describe two alternative transformations as “connected” or “inseparable”, just because they produce contextual correlations. As far as we can tell, the argument for non-locality is based on a literal reading of mathematical concepts, as if they represent ontological categories. (“If one is inseparable, then so must be the other”.) Still, it would be unfair to single out John Bell for this mistake, given that the whole community accepted his argument without consequential resistance. Furthermore, classical waves had been interpreted with pre-existing components long before the discovery of quantum mechanics, and they still are [27]. This practice also makes little physical sense in classical contexts, where real objects cannot move in several directions at the same time. Accordingly, it is worth asking whether any observable phenomenon can be truly “weird”, or whether this is all a consequence of “mathematical realism” in modern physics.
